# Ventricular Electrocardiographic Signatures Associated with Dementia and Plasma Alzheimer’s Disease Biomarkers in Older Adults: A Population-Based Study

**DOI:** 10.3233/JAD-230056

**Published:** 2023-08-15

**Authors:** Ming Mao, Chaoqun Wang, Tingting Hou, Xiaolei Han, Rui Liu, Qi Han, Yi Dong, Jiafeng Wang, Cuicui Liu, Lin Cong, Yume Imahori, Davide Liborio Vetrano, Yongxiang Wang, Yifeng Du, Chengxuan Qiu

**Affiliations:** a Department of Neurology, Shandong Provincial Hospital, Shandong University, Jinan, Shandong, China; b Department of Neurology, Shandong Provincial Hospital affiliated to Shandong First Medical University, Jinan, Shandong, China; c Aging Research Center and Center for Alzheimer Research, Department of Neurobiology, Care Sciences and Society, Karolinska Institutet-Stockholm University, Stockholm, Sweden; d Stockholm Gerontology Research Center, Stockholm, Sweden

**Keywords:** Alzheimer’s disease, dementia, electrocardiogram, neurodegeneration, population-based study

## Abstract

**Background:**

Evidence has emerged that altered ventricular electrocardiogram profiles are associated with dementia, but the neuropathological mechanisms underlying their associations are poorly understood.

**Objective:**

To investigate the interrelationships of ventricular electrocardiogram profiles with dementia and plasma Alzheimer’s disease (AD) biomarkers among older adults.

**Methods:**

This population-based cross-sectional study included 5,153 participants (age ≥65 years; 57.3% women) living in rural communities in China; of these, 1,281 had data on plasma amyloid-β (Aβ)_40_, Aβ_42_, total-tau, and neurofilament light chain (NfL) protein. The QT, QTc, JT, JTc, QRS intervals, and QRS axis were derived from the 10-second electrocardiogram recording. The DSM-IV criteria were followed for clinical diagnosis of dementia, the NIA-AA criteria for AD, and the NINDS-AIREN criteria for vascular dementia (VaD). Data were analyzed using general linear models, multinomial logistic models, and restricted cubic splines.

**Results:**

Of the 5,153 participants, 299 (5.8%) were diagnosed with dementia, including 194 with AD and 94 with VaD. Prolonged QT, QTc, JT, and JTc intervals were significantly associated with all-cause dementia, AD, and VaD (*p* < 0.05). Left QRS axis deviation was significantly associated with all-cause dementia and VaD (*p* < 0.01). In the subsample of plasma biomarkers (*n* = 1,281), prolonged QT, JT, and JTc intervals were significantly associated with a lower Aβ_42_/Aβ_40_ ratio and higher plasma NfL concentrations (*p* < 0.05).

**Conclusion:**

Alterations in ventricular repolarization and depolarization are independently associated with all-cause dementia, AD, VaD, and AD plasma biomarkers in older adults (age ≥65 years). Ventricular electrocardiogram parameters may be valuable clinical markers for dementia and the underlying AD pathologies and neurodegeneration.

## INTRODUCTION

Dementia, as a global public health priority, affects over 55 million people worldwide, with the number being projected to reach 78 million by 2030 [1]. Currently, there is no curative therapy for dementia; however, studies have suggested that interventions targeting the major modifiable risk factors could reduce the global number of people with dementia by ∼40% [2]. Notably, in the past 2-3 decades, evidence has accumulated that cardiovascular risk burden over the lifespan is strongly associated with late-life cognitive impairment and dementia, which supports the view that major cardiovascular risk factors could lead to disorders of the heart, the brain, and the cognition, which are sequentially connected during the aging process [3, 4]. In addition, numerous population-based studies have shown that cardiovascular diseases (CVDs) (e.g., coronary heart disease, heart failure, and atrial fibrillation) are associated with an increased risk of dementia [5–7], partially due to thromboembolism, reduced cardiac output, cerebral hypoperfusion, and cerebral small vessel disease [8].

Electrocardiogram (ECG) is widely used to record cardiac electric activity, which may indicate various underlying clinical and subclinical cardiac disorders [9]. Of particular interest is the possibility to explore ventricular function through QT/JT intervals and QRS interval/QRS axis, which reflect ventricular repolarization and depolarization during each cardiac cycle [10]. JT interval (i.e., QT-QRS) is thought to better represent the specific repolarization time than QT interval [11]. Previous studies have linked prolonged ventricular repolarization with impaired right ventricular function, stroke, and mortality [12, 13]. In addition, a few population-based studies have suggested that some ECG parameters (e.g., high resting heart rate, left ventricular hypertrophy, and prolonged QT interval) are associated with cognitive impairment and dementia in middle-aged and older adults [14]. However, it remained unclear whether the associations between ECG markers and cognitive phenotypes (e.g., dementia, Alzheimer’s disease [AD], and vascular dementia [VaD]) are present independent of major CVDs. This is important because an association of ECG markers with dementia in people without CVDs suggests that these ECG parameters in the preclinical phase of cardiovascular events may be valuable markers for dementia.

Cardiovascular disorders may contribute to late-life cognitive phenotypes possibly via cerebral hypoperfusion and cerebral small vessel disease [3, 4, 8]. It is unclear whether typical AD pathologies (e.g., amyloid-β [Aβ] and tau proteins) are involved in the CVD-dementia association. A clinic-based study detected Aβ aggregates in the heart of patients with a primary diagnosis of AD [15]. Additionally, a case-control study showed that patients with AD but without clinical cardiac disease exhibited electrocardiographic and echocardiographic abnormalities, which appeared to reproduce the pattern of cardiac amyloidosis [16]. These studies suggest the potential that subclinical cardiac disorders in patients with AD might be associated with Aβ deposition. Evidence has emerged that plasma Aβ, tau, and neurofilament light chain (NfL) proteins are correlated with AD pathology or biomarkers in the brain and cerebrospinal fluid [17]. However, no studies have so far examined the association of abnormal ECG parameters with AD-related plasma biomarkers. Thus, exploring their associations may shed light on the underlying neuropathological mechanisms linking clinical and subclinical CVDs with AD and all-cause dementia.

Therefore, we hypothesize that altered ventricular ECG signatures are associated with all-cause dementia, AD, and VaD as well as with plasma biomarkers for AD pathologies and neurodegeneration in older adults (e.g., age ≥65 years). In this large-scale population-based study, we sought to test this hypothesis by assessing the associations of multiple ventricular ECG parameters (i.e., QRS axis and QT, QTc, JT, JTc, and QRS intervals) with all-cause dementia, main subtypes of dementia, and AD plasma biomarkers in a rural population of older adults (age ≥65 years) in China.

## METHODS

### Study design and participants

**Fig. 1:**
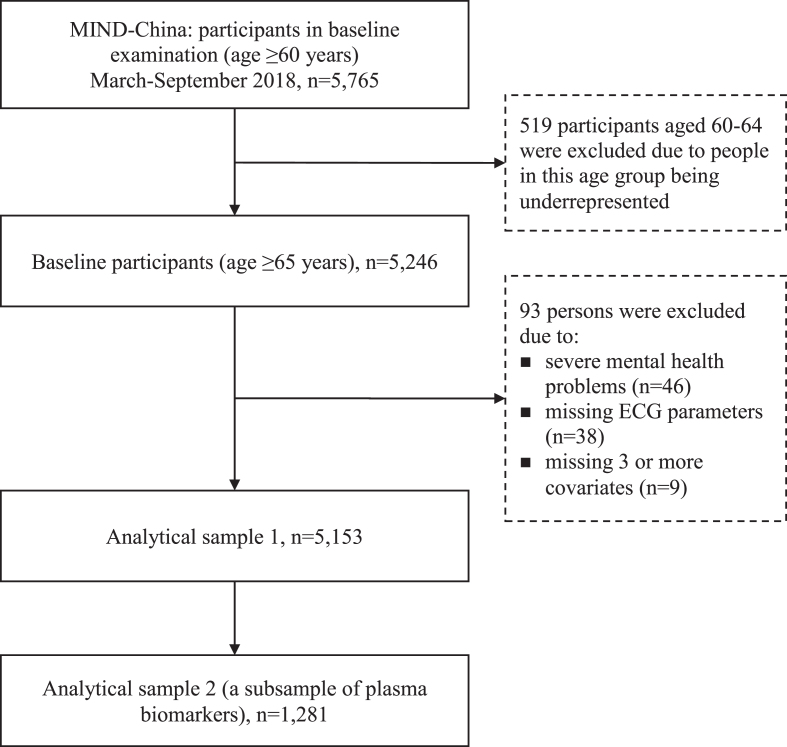
Flowchart of the study participants. MIND-China, Multidomain Interventions to Delay Dementia and Disability in Rural China; ECG, electrocardiogram.

This is a population-based cross-sectional study. Study participants were derived from participants in the baseline assessments of the ongoing Multidomain Interventions to Delay Dementia and Disability in Rural China (MIND-China) study, which is a participating project of the World-Wide FINGERS Network [18]. MIND-China targeted people who were aged 60 years and older and living in the 52 villages (rural communities) of Yanlou town, Yanggu County, western Shandong province, as previously reported [19–21]. Briefly, in March-September 2018, a total number of 5,765 participants (74.9% of all eligible persons) underwent baseline examination. Of these, we excluded 519 participants who were aged 60–64 years because people in this age group were substantially underrepresented due to the fact that a considerable proportion of them were temporarily working as rural migrant workers, and thus, were not available for the examination [22]. Of the remaining 5,246, we further excluded 93 participants due to severe mental health problems (e.g., schizophrenia and major depressive symptoms defined as the 15-item Geriatric Depression Scale score ≥5, *n* = 46), missing ECG parameters (*n* = 38), and missing 3 or more covariates (*n* = 9), leaving 5,153 participants for the analysis involving ECG parameters in association with dementia and subtypes of dementia (analytical sample 1). There were no significant differences between participants in the analytical sample (*n* = 5,153) and those who were excluded (*n* = 93) in mean age (71.75 versus 71.33 years, *p* = 0.524) and distribution of sex (female 57.3% versus 49.5%, *p* = 0.159) and education (no formal education 40.6% versus 44.1%, *p* = 0.765). Out of the 5,153 participants, data on plasma AD and neurodegenerative biomarkers were available in a subsample of 1,281 individuals that consisted of 1,205 participants from 18 villages that were randomly selected from all the 52 villages plus 76 persons with AD who were identified from other villages. This subsample was used for the analysis involving AD plasma biomarkers (analytical sample 2). Figure 1 shows the flowchart of the study participants.

The MIND-China study was approved by the Ethics Committee at Shandong Provincial Hospital in Jinan, Shandong Province, China. Written informed consent was obtained from all participants or in case of participants with severe cognitive impairment, from their guardian or a proxy, usually the family members. This study was conducted in full compliance with the ethical principles outlined in the Declaration of Helsinki. MIND-China was registered in the Chinese Clinical Trial Registry (registration no.: ChiCTR1800017758).

### Data collection and assessments

The trained medical staff collected data following the structured questionnaire *via* face-to-face interviews, clinical and neurological examination, neuropsychological testing, and laboratory tests, as previously described [23]. Demographic features (age, sex, and education), lifestyle factors (smoking and alcohol consumption), apolipoprotein E (*APOE*) gene, health history (e.g., chronic health conditions), and use of drugs (e.g., anti-thrombotic agents, cardiac agents, and QT prolonging drugs) were considered as potential confounders in the analysis. Education was categorized into no formal education, primary school (1–5 years), and middle school or above (≥6 years). Smoking status and alcohol intake were categorized into never, former, and current smoking or alcohol drinking. *APOE* genotype was dichotomized into carriers versus non-carriers of the *ɛ*4 allele. Body mass index (BMI) was calculated as measured weight divided by height squared (kg/m^2^). Information on the current use of medications was collected via in-person interviews, and whenever available, drug prescriptions and containers were checked to verify the information. All medications were classified and coded according to the Anatomical Therapeutic Chemical (ATC) classification system [24]. Diabetes mellitus, dyslipidemia, and hypertension were defined by integrating self-reported history of respective disorders, clinical examination and blood tests (i.e., blood pressure measurement, fasting blood glucose, and serum lipids), and current use of respective mediations (i.e., antihypertensive, blood glucose-lowering, and lipids-lowering drugs), as previously described [22]. Coronary heart disease, heart failure, and arrhythmia were defined as self-reported history of the disease or diagnosis by clinicians based on ECG or medical history. Arrhythmia included atrial fibrillation, premature contractions and other arrhythmia (e.g., supraventricular arrhythmia, junctional rhythms, and intermittent conduct defect). Stroke and transient ischemic attacks (TIAs) were ascertained by self-reported history or diagnosis by neurologists through clinical and neurological examination. We counted the number of common chronic diseases that concurrently occurred in the same participant that included diabetes mellitus, dyslipidemia, hypertension, coronary heart disease, heart failure, arrhythmia, stroke, and TIAs. Global cognitive function was assessed with the Chinese version of the Mini-Mental State Examination (MMSE). Antithrombotic agents (acetylsalicylic acid, clopidogrel, and warfarin), cardiac agents (amiodarone, digoxin, propafenone, isosorbide mononitrate, glyceryl trinitrate, and trimetazidine), and QT prolonging drugs (alprazolam, olanzapine, perphenazine, sulpiride, and tamoxifen) were considered as potential confounders because use of these drugs might affect ECG parameters and might be associated with dementia as well [6, 25].

### Measurements of ECG

The ECG was recorded in a resting supine position using a 10-s 12-lead CM 300 electrocardiograph (Comen Corp., Shenzhen, Guangdong, China) in a local hospital and was analyzed by physicians, following the standard procedure. It was assured that no electrical devices nearby could interfere with electrical signals, and the examining physician helped the participants with cognitive impairment or dementia during the ECG examination. Resting heart rate, QRS interval, QT interval, and QRS axis were derived from an automated analysis program in the device. JT interval was defined as difference between the length of the QT interval and the duration of the QRS complex [26]. QTc and JTc intervals are the heart rate-corrected QT and JT intervals, respectively, which were calculated using the Bazett’s formula [27]: QTc = QT/(60/[heart rate])^1/2^; JTc = JT/(60/[heart rate])^1/2^.

### Diagnosis of dementia, Alzheimer’s disease, and vascular dementia

Dementia was clinically diagnosed by neurologists in accordance with the Diagnostic and Statistical Manual of Mental Disorders, 4th edition (DSM-IV), criteria [28], in which a 3-step diagnostic procedure was followed [20, 21]. Briefly, clinicians and trained medical staff carried out clinical examination and comprehensive assessments following a structured questionnaire, which included health-related factors, medical history, the Chinese version of Activities of Daily Living (ADLs), and a neurocognitive test battery. The neurocognitive test battery was used to assess the global cognitive function and function of four specific cognitive domains: episodic memory, verbal fluency, attention, and executive function, as previously described [29]. Then, neurologists specialized in dementia diagnosis and care reviewed all these records and made a preliminary diagnosis for participants who were suspected to have dementia or who did not have sufficient information for the diagnosis of dementia. Finally, all these persons or their informants or both were interviewed by senior neurologists, and their medical history, cognitive status, ADLs, and whenever available, neuroimaging data, were reassessed. Dementia was further categorized into AD, VaD, and other types of dementia. AD was diagnosed in accordance with the National Institute on Aging-Alzheimer’s Association (NIA-AA) criteria for probable Alzheimer’s dementia [30], and VaD was diagnosed following the National Institute of Neurological Disorders and Stroke and the Association Internationale pour la Recherche et l’Enseignement en Neurosciences (NINDS-AIREN) criteria for probable VaD [31]. Dementia cases who could not be classified as either AD or VaD were considered to have other types of dementia.

### Measurements of plasma AD biomarkers

Peripheral blood samples were collected into EDTA-coated vacutainers and centrifuged to acquire plasma, and then the plasma samples were aliquoted and stored at –80°C for future analysis, as previously described [32]. Plasma biomarkers for AD and neurodegeneration were detected on a single molecule array (SIMOA) platform (Quanterix Corp, MA, USA) following the manufacturer’s instructions. Plasma Aβ_42_, Aβ_40_, and total tau (t-tau) were measured using the Human Neurology 3-Plex Assay (N3PA), and NfL protein was measured using the NF-light^ ®^ advantage kit. For each analyte, two quality control samples were carried out and the within-batch and inter-batch CVs were <13% [32].

### Statistical analysis

We reported mean (standard deviation, SD) for continuous variables, and frequency (%) for categorical variables. We compared the characteristics of the study participants by dementia status using chi-squared test for categorical variables and Mann-Whitney U test for continuous variables with skewed distribution. ECG parameters were analyzed as both continuous and categorical variables. We calculated Spearman’s correlation coefficient to assess the correlation of QTc and JTc intervals with MMSE score. We implemented logistic regression models to estimate the associations of QT, QTc, JT, JTc, and QRS intervals with all-cause dementia, AD, and VaD, in which these ECG parameters were categorized into quartiles, with the first quartile being the reference group. We tested statistical interaction of the ECG parameters with age strata (<75 versus ≥75 years) and sex by adding independent variables and their multiplicative term into the same model. To examine the relationship of QRS axis with the likelihoods of all-cause dementia, AD, and VaD, we used restricted cubic splines with the median as the reference. Additionally, the general linear regression models were employed to investigate the associations of ventricular ECG parameters with plasma biomarkers for AD and neurodegeneration, in which the standardized QT, QTc, JT, JTc, and QRS intervals were used [26]. Owing to skewed distributions, plasma Aβ_40_ and NfL concentrations were transformed with the natural logarithm. The Aβ_42_/Aβ_40_ ratio was multiplied by 100.

We reported the main results from the models that were adjusted for age, sex, education, smoking, alcohol intake, BMI, *APOE* genotype, resting heart rate (QT, JT, and QRS intervals only), the number of chronic diseases, and use of anti-thrombotic agents, cardiac agents, and QT prolonging drugs. Individuals with missing values for covariables were represented by creating a dummy variable. In the sensitivity analyses, to assess the impact of cardiovascular disorders on the results, we repeated analyses by excluding participants with CVDs (i.e., coronary heart disease, heart failure, and arrhythmia). A two-tailed *p* < 0.05 was considered statistically significant. R version 3.6.2 (R Project for Statistical Computing; http://www.r-project.org) and GraphPad Prism 8.0.2 software were used for all statistical analyses and figure preparations.

## RESULTS

### Characteristics of the study participants

Of the 5,153 participants, 299 (5.8%) were diagnosed with dementia, including 194 with AD, 94 with VaD, and 11 with other subtypes of dementia. The mean age of the total sample was 71.75 (SD = 5.5) years, 57.3% were female, and 40.6% had no formal education (Table 1). Compared to non-demented individuals (*n* = 4,854), people with AD or VaD had longer QTc and JTc intervals and smaller QRS axis angle (all *p* < 0.05). Participants with AD were older, more likely to be female, less educated, and had a lower BMI, a higher prevalence of CHD, and higher resting heart rate (*p* < 0.05). Individuals with VaD were older, less educated, and had more chronic diseases and a higher prevalence of diabetes, dyslipidemia, and stroke (all *p* < 0.05, Table 1).

**Table 1:** Characteristics of study participants by dementia status

Characteristics	Total sample	Dementia status		
	(*n* = 5,153)	No (*n* = 4,854)	AD (*n* = 194)	VaD (*n* = 94)
Age, y	71.75 (5.5)	71.45 (5.3)	77.65 (7.2) ^b^	74.56 (6.2) ^b^
Female sex, *n* (%)	2,953 (57.3)	2,747 (56.6)	146 (75.3) ^b^	54 (57.4)
Educational level, *n* (%)				
No formal education	2,093 (40.6)	1,891 (39.0)	144 (74.2) ^b^	54 (57.4) ^b^
Primary school	2,233 (43.3)	2,157 (44.4)	40 (20.6)	30 (31.9)
Middle school or above	827 (16.0)	806 (16.6)	10 (5.2)	10 (10.6)
Smoking status, *n* (%)				
Never	3,335 (64.7)	3,108 (64.0)	158 (81.4) ^b^	62 (66.0)
Former	767 (14.9)	728 (15.0)	18 (9.3)	19 (20.2)
Current	1,051 (20.4)	1,018 (21.0)	18 (9.3)	13 (13.8)
Alcohol intake^a^, *n* (%)				
Never	3,202 (62.8)	2,970 (61.8)	157 (81.8) ^b^	69 (74.2) ^b^
Former	488 (9.6)	458 (9.5)	12 (6.3)	16 (17.2)
Current	1,411 (27.7)	1,378 (28.7)	23 (12.0)	8 (8.6)
Body mass index, kg/m^2a^	24.83 (3.8)	24.86 (3.8)	23.72 (3.9) ^b^	25.66 (4.5)
Number of chronic diseases^a^	1.61 (1.2)	1.59 (1.1)	1.69 (1.2)	2.61 (1.3) ^b^
Diabetes mellitus, *n* (%)	735 (14.3)	675 (13.9)	30 (15.5)	28 (29.8) ^b^
Dyslipidemia^a^, *n* (%)	1,226 (23.8)	1,138 (23.4)	51 (26.3)	36 (38.7) ^b^
Hypertension^a^, *n* (%)	3,440 (67.3)	3,240 (67.3)	127 (66.1)	68 (72.3)
Coronary heart disease, *n* (%)	1,131 (21.9)	1,049 (21.6)	55 (28.4) ^b^	26 (27.7)
Heart failure, *n* (%)	148 (2.9)	142 (2.9)	5 (2.6)	1 (1.1)
Arrhythmia, *n* (%)	713 (13.8)	669 (13.8)	29 (14.9)	15 (16.0)
Stroke, *n* (%)	820 (15.9)	719 (14.8)	30 (15.5)	68 (72.3) ^b^
Transient ischemic attacks, *n* (%)	57 (1.1)	53 (1.1)	2 (1.0)	2 (2.1)
Anti-thrombotic agents, *n* (%)	353 (6.9)	325 (6.7)	8 (4.1)	20 (21.3) ^b^
Cardiac agents, *n* (%)	84 (1.6)	78 (1.6)	3 (1.5)	3 (3.2)
QT prolonging drugs, *n* (%)	6 (0.1)	6 (0.1)	0 (0)	0 (0)
*APOE* *ɛ*4 allele^a^, *n* (%)	794 (16.0)	745 (15.9)	34 (18.7)	14 (17.1)
Resting heart rate, bpm	67.59 (11.2)	67.45 (11.1)	69.25 (10.6) ^b^	71.13 (13.0)
QT interval length, ms	397.35 (31.5)	397.24 (31.3)	400.84 (33.1)	396.32 (33.4) ^b^
QTc interval length, ms	418.52 (26.0)	417.98 (25.7)	427.68 (28.5) ^b^	427.74 (27.8) ^b^
JT interval length, ms	300.00 (32.1)	299.77 (32.0)	305.19 (33.1) ^b^	300.81 (32.3)
JTc interval length, ms	315.60 (25.8)	315.04 (25.6)	325.22 (26.9) ^b^	324.38 (27.4) ^b^
QRS interval length, ms	97.36 (12.7)	97.47 (12.7)	95.65 (13.0) ^b^	95.51 (12.5)
QRS axis, °	37.60 (39.0)	38.03 (38.8)	34.28 (39.5)^b^	21.97 (42.2)^b^

Data are mean (standard deviation), unless otherwise specified. AD, Alzheimer’s disease; VaD, vascular dementia; *APOE*, apolipoprotein E gene. ^a^The number of participants with missing values was 192 for *APOE* genotype, 28 for body mass index, 52 for alcohol intake, 1 for dyslipidemia, 42 for hypertension, and totally 43 for the number of chronic diseases. In subsequent analyses, participants with missing values for each of these variables were replaced with a dummy variable. ^b^*p* < 0.05 for the comparison with non-dementia group.

### Associations of ventricular ECG profiles with dementia and its subtypes (n = 5,153)

The multivariable-adjusted odds ratios (OR) of all-cause dementia associated with QT interval quartiles (Q1–Q4) were 1.00 (reference), 1.67 (95% confidence interval [CI]: 1.13, 2.50), 1.93 (1.26, 2.97), and 1.73 (1.08, 2.79), respectively (p for trend = 0.02). QTc, JT, and JTc intervals had analogous associations with all-cause dementia (Table 2). These ventricular ECG parameters were significantly associated with an increased likelihood of AD and VaD, but the association with VaD appeared to be stronger than that with AD. In addition, QRS interval was not significantly associated with all-cause dementia, AD, and VaD (Table 2). We did not detect any statistical interaction of these ECG parameters with age strata and sex on the likelihood of dementia and subtypes of dementia (p for all interactions >0.17). There was a significant correlation between QTc and JTc intervals with MMSE score (Spearman’s correlation coefficient *r* = –0.20, *p* < 0.001, *r* = –0.24, *p* < 0.001, respectively).

**Table 2:** Associations of electrocardiographic profiles with all-cause dementia, Alzheimer’s disease, and vascular dementia (*n* = 5,153)

Electrocardiogram	No. of	All-cause dementia		Alzheimer’s disease		Vascular dementia	
parameters	subjects	*n*	Odds ratio (95%	*n*	Odds ratio (95%	*n*	Odds ratio (95%
			confidence interval) ^a^		confidence interval)^a^		confidence interval)^a^
QT interval (quartiles, ms)							
Q1 (<377)	1,275	65	1.00 (reference)	40	1.00 (reference)	21	1.00 (reference)
Q2 (377–396)	1,288	78	1.67 (1.13, 2.50)^ *^	46	1.45 (0.88, 2.41)	28	2.44 (1.25, 4.89)^ *^
Q3 (396–416)	1,291	81	1.93 (1.26, 2.97)^ †^	58	1.91 (1.14, 3.27)^ *^	23	2.53 (1.20, 5.44)^ *^
Q4 (≥416)	1,299	75	1.73 (1.08, 2.79)^ *^	50	1.55 (0.86, 2.82)	22	2.28 (1.01, 5.27)^ *^
*p* for trend			0.02		0.10		0.06
QTc interval (quartiles, ms)							
Q1 (<402)	1,287	40	1.00 (reference)	26	1.00 (reference)	13	1.00 (reference)
Q2 (402–416)	1,285	64	1.51 (1.00, 2.31)	43	1.40 (0.85, 2.36)	17	1.50 (0.71, 3.23)
Q3 (416–432)	1,292	91	1.88 (1.27, 2.82)^ †^	59	1.64 (1.01, 2.71)	30	2.29 (1.18, 4.68)^ *^
Q4 (≥432)	1,289	104	1.70 (1.15, 2.56)^ †^	66	1.44 (0.89, 2.40)	34	2.07 (1.07, 4.24)^ *^
*p* for trend			0.005		0.11		0.02
JT interval (quartiles, ms)							
Q1 (<279)	1,245	58	1.00 (reference)	39	1.00 (reference)	16	1.00 (reference)
Q2 (279–298)	1,266	76	1.62 (1.08, 2.45)^ *^	43	1.06 (0.64, 1.77)	31	3.74 (1.84, 7.97)^ ‡^
Q3 (298–319)	1,335	87	2.00 (1.30, 3.10)^ †^	59	1.52 (0.91, 2.59)	25	3.63 (1.65, 8.33)^ †^
Q4 (≥319)	1,307	78	1.73 (1.07, 2.83)^ *^	53	1.19 (0.66, 2.17)	22	3.45 (1.45, 8.53)^ †^
*p* for trend			0.02		0.35		0.009
JTc interval (quartiles, ms)							
Q1 (<299)	1,282	37	1.00 (reference)	25	1.00 (reference)	11	1.00 (reference)
Q2 (299–314)	1,288	66	1.69 (1.11, 2.60)^ *^	40	1.39 (0.83, 2.37)	22	2.07 (1.00, 4.53)
Q3 (314–331)	1,294	86	1.98 (1.32, 3.04)^ †^	57	1.67 (1.02, 2.81)^ *^	27	2.59 (1.27, 5.66)^ *^
Q4 (≥331)	1,289	110	2.12 (1.41, 3.25)^ ‡^	72	1.66 (1.02, 2.79)^ *^	34	3.05 (1.50, 6.64)^ †^
*p* for trend			<0.001		0.03		0.003
QRS interval (quartiles, ms)							
Q1 (<90)	1,276	88	1.00 (reference)	57	1.00 (reference)	26	1.00 (reference)
Q2 (90–96)	1,183	78	1.15 (0.82, 1.60)	48	1.12 (0.74, 1.69)	28	1.32 (0.75, 2.34)
Q3 (96–103)	1,374	67	0.85 (0.60, 1.20)	52	1.11 (0.74, 1.67)	15	0.57 (0.29, 1.11)
Q4 (≥103)	1,320	66	1.01 (0.70, 1.44)	37	1.03 (0.65, 1.63)	25	0.93 (0.51, 1.72)
*p* for trend			0.63		0.91		0.29

^a^Models were adjusted for age, sex, education, resting heart rate (QT, JT and QRS intervals only), APOE genotype, smoking status, alcohol intake, body mass index, the number of chronic diseases, and use of anti-thrombotic agents, cardiac agents, and QT prolonging drugs. ^*^p < 0.05, ^†^*p* < 0.01, ^‡^*p* < 0.001.

We repeated the aforementioned analyses by excluding 1,660 participants with CVDs (i.e., coronary heart disease, heart failure, and arrhythmia), which yielded the results that were overall similar to those reported in Table 2 (Supplementary Table 1).

**Fig. 2:**
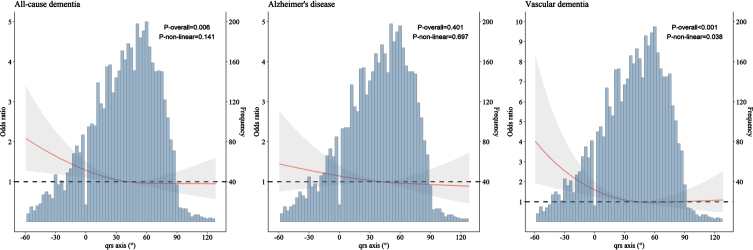
Association of QRS axis with all-cause dementia, Alzheimer’s disease, and vascular dementia derived from restricted cubic spline models (*n* = 5,153). Restricted cubic spline models were adjusted for age, sex, education, *APOE* genotype, smoking status, alcohol intake, body mass index, the number of chronic diseases, and use of anti-thrombotic agents, cardiac agents, and QT prolonging drugs.

We carried out restricted cubic splines modeling analysis to examine the patterns of association of QRS axis with all-cause dementia, AD, and VaD. The results suggested a J-shaped pattern of association, such that left-deviated QRS axis (–90°, –30°) was associated with an elevated likelihood of having dementia (*p* = 0.006, p for non-linearity = 0.141) and VaD (*p* < 0.001, p for non-linearity = 0.038), while right deviation (90°, 180°) was not. QRS axis deviation was not significantly associated with AD (Fig. 2). Similar results were obtained when excluding participants with CVDs from the analysis (Supplementary Figure 1).

### Associations of ventricular ECG profiles with AD plasma biomarkers (n = 1,281)

**Fig. 3:**
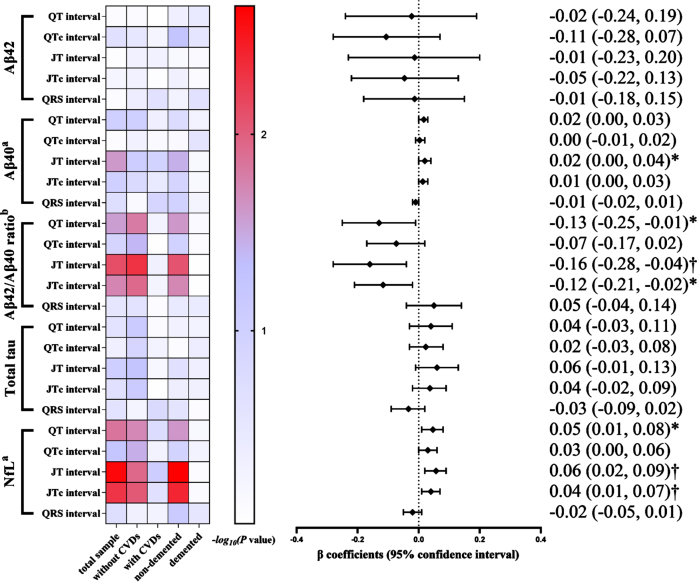
Association of electrocardiographic parameters with Alzheimer’s disease plasma biomarkers in the total biomarker subsample and by cardiovascular disease and Alzheimer’s disease status (*n* = 1,281). Aβ, amyloid-β; NfL, neurofilament light chain protein; CVDs, cardiovascular diseases. The heatmap indicated *p* value for association of ECG profiles with plasma biomarkers among all participants in the total biomarker subsample (*n* = 1,281), in participants free of cardiovascular disease (*n* = 891) and those with cardiovascular disease (*n* = 390), and in participants without dementia (*n* = 1,139) and those with dementia (*n* = 142). The results on the right are from the total biomarker subsample. Models were adjusted for age, sex, education, resting heart rate (QT, JT and QRS intervals only), *APOE* genotype, smoking status, alcohol intake, body mass index, the number of chronic diseases, and use of anti-thrombotic agents, cardiac agents, and QT prolonging drugs. ^a^The original data of the plasma Aβ_40_ and NfL concentrations were natural log-transformed due to skewed distributions. ^b^Plasma Aβ_42_/Aβ_40_ ratio was multiplied by 100. ^ *^p < 0.05, ^ †^*p* < 0.01.

In the subsample of plasma biomarkers (*n* = 1,281), an increased JT interval was significantly correlated with a higher plasma Aβ_40_ level (*p* < 0.05), whereas none of the ventricular ECG parameters was associated with plasma Aβ_42_. Additionally, the multivariable-adjusted β coefficients of plasma Aβ_42_/Aβ_40_ ratio associated with QT, JT, and JTc intervals were –0.13 (95% CI: –0.25, –0.01), –0.16 (–0.28, –0.04), and –0.12 (–0.21, –0.02), respectively. The multivariable-adjusted β coefficients of plasma NfL concentration associated with QT, JT, and JTc intervals were 0.05 (0.01, 0.08), 0.06 (0.02, 0.09), and 0.04 (0.01, 0.07), respectively (Fig. 3). The analyses stratified by CVD or dementia yielded results among participants without CVDs (*n* = 891) or those without dementia (*n* = 1,139) that were similar to those in the total sample, whereas no significant associations were found in participants who had clinical CVDs (*n* = 390) or dementia (*n* = 142) (Fig. 3, Supplementary Table 2).

## DISCUSSION

In this population-based study of rural Chinese older adults, we investigated the associations of ECG profiles with dementia and several plasma biomarkers for AD and neurodegeneration. We found that prolonged ventricular repolarization was associated with an increased likelihood of all-cause dementia, AD, and VaD, as well as with elevated plasma Aβ_40_ and NfL concentrations and a decreased Aβ_42_/Aβ_40_ ratio. In addition, altered depolarizing direction was associated with dementia, especially VaD. Notably, these associations remained even among participants without clinical CVDs. Taken together, our study suggests that ventricular ECG signatures may be valuable clinical markers for dementia and that both conditions may share common AD and neurodegenerative pathology.

A systematic review found mixed results in the current literature with regard to the associations of ventricular ECG profiles with dementia and cognitive performance in middle-aged and older adults, partly due to methodological heterogeneity, which emphasized the need of further investigation [14]. For example, the Prospective Study of Pravastatin in the Elderly at Risk among individuals who were free of cognitive impairment and arrhythmia showed that prolonged ventricular repolarization was cross-sectionally associated with poor cognitive performance [26]. Besides, a cross-sectional study of community-living older adults in Italy supported the association of QTc dispersion (a metric of ventricular repolarization) with mild cognitive impairment and AD [33]. Furthermore, left-deviated QRS axis is related to left ventricular hypertrophy (LVH), and the Atherosclerosis Risk in Communities-Neurocognitive Study of middle-aged adults revealed a cross-sectional association between LVH and poor cognitive performance [34]. These studies are in line with our findings that prolonged intervals of QT, QTc, JT, and JTc and left-deviated QRS axis were associated with dementia and the main subtypes of dementia. By contrast, the Chicago Health and Aging Project (59% blacks) found no association of QTc interval with global cognitive performance [35]. Notably, we extended previous findings by showing that the associations of prolonged ventricular repolarization and left-deviated QRS axis with all-cause dementia and subtypes of dementia were present, independent of clinical CVDs. This indicates that altered ventricular ECG profiles, measured even at preclinical phase of CVDs, may be valuable markers for dementia. Indeed, the prolonged QTc and JTc intervals and left-deviated QRS axis have been associated with an elevated risk of incident coronary heart disease and heart failure [11, 36]. Taken together, these findings suggest that certain ventricular ECG parameters may be valuable markers for dementia in olderadults.

This is the first population-based study that linked prolonged ventricular repolarization with increased plasma Aβ_40_ and NfL concentrations and a decreased Aβ_42/40_ ratio in older adults. Evidence has emerged that plasma Aβ_42/40_ ratio may reflect amyloidosis in central nervous system [17]. The clinical-based amyloid-PET studies showed that plasma Aβ_42/40_ ratio could be used for the diagnosis of brain amyloidosis, even among cognitively normal individuals [37], but the ratio was not strongly associated with clinical diagnosis of AD [38]. Notably, our results showed that ventricular ECG parameters remained associated with a low plasma Aβ_42/40_ ratio in participants without clinical dementia. This suggests that altered ventricular ECG profiles might be linked to the underlying AD pathologies. Besides, an autopsy-verified study reported that patients with a primary diagnosis of AD had Aβ aggregates in their heart [15], and a case-control study found subclinical cardiac changes (e.g., abnormal ECG parameters) among patients with AD [16]. Collectively, these studies indicate that subclinical cardiac conditions might be associated with cardiac Aβ aggregates. Furthermore, Aβ deposits (especially Aβ_1 - 40_ peptide) in cerebral microvasculature lead to cerebral amyloid angiopathy, which has been associated with vascular cognitive impairment [39]. Indeed, our results showed that altered ventricular ECG profiles were associated with both plasma Aβ_40_ and VaD. We also found that prolonged ventricular repolarization was associated with an elevated plasma NfL concentration. NfL is a cytoskeleton protein released into the extracellular fluid as a consequence of axonal damage [40]. An increased plasma NfL level was associated with autonomic failure [41], and QTc dispersion and QT interval variability were previously linked to autonomous dysfunction [33]. Thus, prolonged QT and JT intervals may be manifestations of autonomic dysfunction, which is common in patients with AD and other neurodegenerative disorders [42, 43]. Additionally, an elevated plasma NfL concentration was correlated with several risk factors for dementia, such as hypertension, dyslipidemia, and diabetes [40]. Of note, our study showed that the associations of abnormal ECG parameters with reduced plasma Aβ_42_/Aβ_40_ ratio and increased NfL concentrations remained in participants without clinical CVDs or in individuals free of clinical dementia. Taken together, these findings indicate that abnormal ventricular ECG parameters and dementia may share common AD and neurodegenerative pathologies in olderadults.

This is a large-scale population-based study that targeted older adults living in rural communities in China where people had relatively low income and very limited education; this sociodemographic group has been substantially underrepresented in dementia research. In addition, we were able to explore the potential mechanisms underlying the associations of ECG parameters with cognitive outcomes in a subsample where comprehensive epidemiological and clinical data were integrated with plasma biomarkers assessed using the state-of-the-art SIMOA technology. However, our study has limitations. First, due to the cross-sectional nature of the study, we could not determine the causal relationship of ventricular ECG signatures with dementia and plasma biomarkers. Second, our ventricular ECG parameters were derived from the one-time 10-s ECG recording. Previous studies have shown that the ECG parameters from the single 10-s ECG are less accurate in reflecting the cardiac conditions compared to those from the multiple 10-s ECG or 5-min ECG recording, especially in people with cardiovascular disorders (e.g., atrial fibrillation) [44, 45]. Third, despite the adjustment for a range of potential confounders, our results might still be affected by residual confounding due to imperfect assessments of some confounders (e.g., self-reported lifestyle factors and arrhythmia that may not be accurately detected by self-reported history and the 10-s ECG recording). Finally, the study participants were recruited from only one rural region in western Shandong province. Thus, cautiousness is needed when generalizing our results to otherpopulations.

In conclusion, this population-based study revealed that ECG markers of prolonged ventricular repolarization were associated with all-cause dementia, AD, and VaD among older adults, independent of clinical CVDs. Furthermore, altered depolarizing direction was associated with dementia, and VaD in particular. Finally, ECG markers for prolonged ventricular repolarization were associated with plasma AD and neurodegenerative biomarkers. These results suggest that the ECG parameters may be useful clinical markers for dementia and that altered ECG signatures and dementia may share common AD and neurogenerative pathology in older adults.

## Supplementary Material

Supplementary Material

## Data Availability

The datasets used and/or analyzed during the current study are available from the corresponding author upon reasonable request.
