# Supplementary material for: Ventricular Electrocardiographic Signatures Associated with Dementia and Plasma Alzheimer’s Disease Biomarkers in Older Adults: A Population-Based Study
Source: J Alzheimers Dis. 2023 Aug 15;94(4):1515–26. doi: 10.3233/JAD-230056 (PMC10473117; doi:10.3233/JAD-230056)
Supplement: Supplementary Material [file jad-94-jad230056-s001.pdf]

SUPPLEMENTARY MATERIAL

Ventricular electrocardiographic signatures associated with dementia and plasma Alzheimer's biomarkers in older adults: a population-based study

Ming Mao, MD,[1] Chaoqun Wang, MD,[1] Tingting Hou, MD, PhD,[2] Xiaolei Han, MD, PhD,[2] Rui Liu, MD,[1] Qi Han, MD,[1] Yi Dong, MD, PhD,[2] Jiafeng Wang,[1] MD; Cuicui Liu, MD,[2] Lin Cong, MD, PhD,[2] Yume Imahori, MD, PhD,[3] Davide Liborio Vetrano, MD, PhD,[3,4] Yongxiang Wang, MD, PhD,[2,3*] Yifeng Du, MD, PhD,[1,2*] Chengxuan Qiu, PhD[1,3]

[1]Department of Neurology, Shandong Provincial Hospital, Shandong University, Jinan, Shandong, China;
[2]Department of Neurology, Shandong Provincial Hospital affiliated to Shandong First Medical University, Jinan, Shandong, China;
[3]Aging Research Center and Center for Alzheimer Research, Department of Neurobiology, Care Sciences and Society, Karolinska Institutet-Stockholm University, Stockholm, Sweden;
[4]Stockholm Gerontology Research Center, Stockholm, Sweden.

CONTENTS

Supplemental Figure

1. **Supplementary Figure 1.** Associations of QRS axis with all-cause dementia, Alzheimer's disease, and vascular dementia among participants without cardiovascular diseases derived from restricted cubic spline models (n=3,493)

Supplemental Tables

1. **Supplementary Table 1.** Associations of electrocardiographic profiles with all-cause dementia, Alzheimer's disease, and vascular dementia among participants without cardiovascular diseases (n=3,493)
2. **Supplementary Table 2.** Associations of electrocardiographic profiles with Alzheimer's disease plasma biomarkers by cardiovascular diseases and dementia status

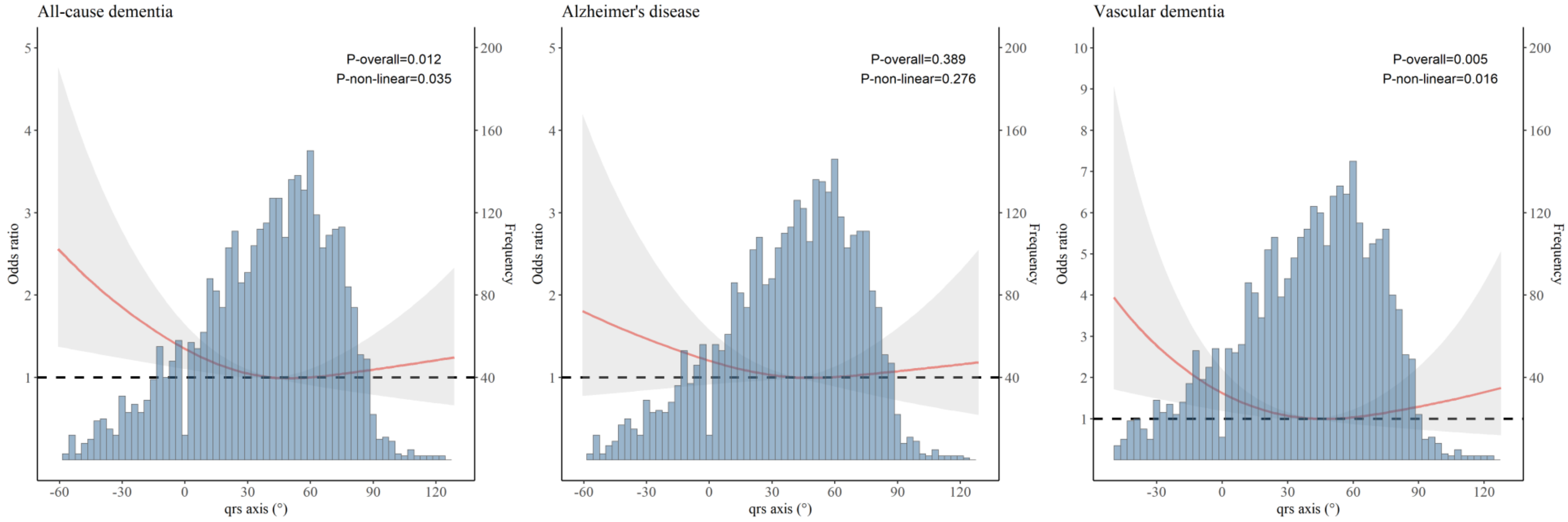

Supplementary Figure 1. Associations of QRS axis with all-cause dementia, Alzheimer's disease, and vascular dementia among participants without cardiovascular disease derived from restricted cubic spline models (n=3,493)

Restricted cubic spline models were adjusted for age, sex, education, *APOE* genotype, smoking status, alcohol intake, body mass index, the number of chronic diseases, and use of anti-thrombotic agents, cardiac agents, and QT prolonging drugs.

Supplementary Table 1. Associations of electrocardiographic profiles with all-cause dementia, Alzheimer's disease, and vascular dementia among participants without cardiovascular disease (n=3,493)

Electrocardiogram parameters	No. of subjects	All-cause dementia		Alzheimer's disease		Vascular dementia	
		n	Odds ratio (95% confidence interval)[a]	n	Odds ratio (95% confidence interval)[a]	n	Odds ratio (95% confidence interval)[a]
**QT interval** (quartiles, ms)							
Q1 (<377)	851	43	1.00 (reference)	26	1.00 (reference)	13	1.00 (reference)
Q2 (377-396)	917	51	1.52 (0.93, 2.50)	29	1.31 (0.70, 2.47)	19	2.63 (1.11, 6.49)*
Q3 (396-416)	888	57	1.96 (1.15, 3.37)*	39	1.98 (1.02, 3.90)*	18	3.57 (1.37, 9.73)*
Q4 (≥416)	837	41	1.64 (0.88, 3.07)	28	1.61 (0.75, 3.49)	10	2.79 (0.86, 9.22)
P for trend			0.08		0.14		0.07
**QTc interval** (quartiles, ms)							
Q1 (<402)	920	27	1.00 (reference)	17	1.00 (reference)	9	1.00 (reference)
Q2 (402-416)	935	45	1.47 (0.89, 2.46)	30	1.45 (0.78, 2.77)	11	1.27 (0.50, 3.29)
Q3 (416-432)	881	58	1.67 (1.03, 2.77)*	35	1.39 (0.76, 2.65)	21	2.43 (1.08, 5.91)*
Q4 (≥432)	757	62	1.65 (1.01, 2.76)*	40	1.45 (0.79, 2.75)	19	2.31 (0.97, 5.85)
P for trend			0.04		0.28		0.03
**JT interval** (quartiles, ms)							
Q1 (<279)	811	37	1.00 (reference)	25	1.00 (reference)	9	1.00 (reference)
Q2 (279-298)	869	48	1.63 (0.98, 2.74)	26	1.00 (0.53, 1.91)	20	5.35 (2.10, 14.79)‡
Q3 (298-319)	951	64	2.15 (1.27, 3.72)†	44	1.62 (0.86, 3.12)	18	5.97 (2.12, 18.21)†
Q4 (≥319)	862	43	1.73 (0.92, 3.28)	27	1.05 (0.49, 2.30)	13	7.06 (2.14, 25.04)†
P for trend			0.06		0.61		0.002
**JTc interval** (quartiles, ms)							
Q1 (<299)	881	24	1.00 (reference)	15	1.00 (reference)	8	1.00 (reference)
Q2 (299-314)	914	42	1.47 (0.87, 2.53)	26	1.32 (0.69, 2.64)	12	1.39 (0.54, 3.72)
Q3 (314-331)	880	58	1.82 (1.10, 3.09)*	36	1.52 (0.81, 2.98)	20	2.69 (1.13, 6.97)*
Q4 (≥331)	818	68	2.04 (1.22, 3.48)†	45	1.69 (0.91, 3.31)	20	3.01 (1.22, 8.02)*
P for trend			0.005		0.09		0.008
**QRS interval** (quartiles, ms)							
Q1 (<90)	885	59	1.00 (reference)	35	1.00 (reference)	20	1.00 (reference)
Q2 (90-96)	832	54	1.07 (0.71, 1.60)	35	1.31 (0.79, 2.18)	17	0.73 (0.35, 1.49)
Q3 (96-103)	934	43	0.78 (0.50, 1.19)	32	1.12 (0.66, 1.89)	11	0.45 (0.20, 0.98)*
Q4 (≥103)	842	36	0.93 (0.58, 1.48)	20	1.08 (0.58, 1.97)	12	0.62 (0.27, 1.37)
P for trend			0.48		0.94		0.14

[a] Models were adjusted for age, sex, education, resting heart rate (QT, JT and QRS intervals only), *APOE* genotype, smoking status, alcohol intake, body mass index, the number of chronic diseases, and use of anti-thrombotic agents, cardiac agents, and QT prolonging drugs.

**P*<0.05, † *P*<0.01, ‡ *P*<0.001.

Supplementary Table 2. Associations of electrocardiographic profiles with Alzheimer's disease plasma biomarkers by cardiovascular disease and dementia status (n=1,281)

ECG parameters	β coefficient (95% confidence interval) [c], plasma biomarkers			
	Cardiovascular diseases		Dementia	
	No (n=891)	Yes (n=390)	No (n=1139)	Yes (n=142)
**Aβ42 (pg/ml)**				
QT interval	-0.04 (-0.29, 0.22)	0.01 (-0.39, 0.41)	-0.08 (-0.30, 0.13)	0.44 (-0.48, 1.36)
QTc interval	-0.10 (-0.30, 0.10)	-0.08 (-0.42, 0.25)	-0.17 (-0.34, 0.01)	0.39 (-0.32, 1.09)
JT interval	-0.08 (-0.33, 0.17)	0.14 (-0.27, 0.56)	-0.04 (-0.26, 0.17)	0.16 (-0.75, 1.08)
JTc interval	-0.07 (-0.27, 0.13)	0.03 (-0.32, 0.38)	-0.06 (-0.24, 0.11)	0.10 (-0.62, 0.83)
QRS interval	0.08 (-0.11, 0.27)	-0.20 (-0.54, 0.14)	-0.05 (-0.23, 0.12)	0.42 (-0.29, 1.13)
**Aβ40 (pg/ml)[a]**				
QT interval	0.02 (0.00, 0.04)	0.01 (-0.02, 0.04)	0.01 (-0.01, 0.03)	0.02 (-0.04, 0.08)
QTc interval	0.01 (-0.01, 0.02)	0.00 (-0.03, 0.02)	0.00 (-0.02, 0.01)	0.03 (-0.02, 0.07)
JT interval	0.02 (0.00, 0.04)	0.03 (-0.01, 0.06)	0.02 (0.00, 0.04) *	0.01 (-0.05, 0.07)
JTc interval	0.01 (-0.01, 0.03)	0.01 (-0.01, 0.04)	0.01 (0.00, 0.03)	0.01 (-0.04, 0.06)
QRS interval	0.00 (-0.02, 0.01)	-0.02 (-0.05, 0.01)	-0.01 (-0.03, 0.00)	0.01 (-0.03, 0.06)
**Aβ42/Aβ40[b]**				
QT interval	-0.18 (-0.33, -0.03) *	-0.06 (-0.26, 0.14)	-0.14 (-0.27, -0.02) *	0.07 (-0.30, 0.43)
QTc interval	-0.12 (-0.24, 0.00) *	0.00 (-0.16, 0.17)	-0.08 (-0.18, 0.02)	0.04 (-0.24, 0.32)
JT interval	-0.21 (-0.35, -0.06) †	-0.08 (-0.28, 0.13)	-0.17 (-0.29, -0.04) †	-0.02 (-0.39, 0.35)
JTc interval	-0.15 (-0.27, -0.03) *	-0.05 (-0.22, 0.13)	-0.12 (-0.23, -0.02) *	-0.01 (-0.30, 0.27)
QRS interval	0.06 (-0.05, 0.18)	0.02 (-0.15, 0.19)	0.05 (-0.05, 0.15)	0.13 (-0.16, 0.41)
**Total tau (pg/ml)**				
QT interval	0.08 (-0.01, 0.16)	-0.02 (-0.14, 0.10)	0.02 (-0.05, 0.10)	0.08 (-0.17, 0.33)
QTc interval	0.05 (-0.01, 0.12)	-0.03 (-0.13, 0.07)	0.01 (-0.05, 0.06)	0.08 (-0.11, 0.28)
JT interval	0.08 (0.00, 0.17)	0.03 (-0.10, 0.15)	0.04 (-0.03, 0.12)	0.09 (-0.16, 0.35)
JTc interval	0.06 (-0.01, 0.13)	-0.01 (-0.11, 0.10)	0.02 (-0.04, 0.08)	0.07 (-0.13, 0.27)
QRS interval	-0.02 (-0.08, 0.05)	-0.07 (-0.18, 0.03)	-0.03 (-0.09, 0.02)	-0.02 (-0.22, 0.18)
**NfL (pg/ml)[a]**				
QT interval	0.05 (0.01, 0.10) *	0.04 (-0.02, 0.10)	0.04 (0.01, 0.08) *	0.03 (-0.11, 0.16)
QTc interval	0.04 (0.00, 0.07) *	0.02 (-0.03, 0.07)	0.02 (-0.01, 0.05)	0.03 (-0.07, 0.13)
JT interval	0.06 (0.01, 0.10) *	0.06 (-0.01, 0.12)	0.06 (0.02, 0.10) †	-0.01 (-0.14, 0.12)
JTc interval	0.05 (0.01, 0.08) †	0.03 (-0.02, 0.09)	0.04 (0.01, 0.08) †	-0.02 (-0.12, 0.09)
QRS interval	-0.01 (-0.05, 0.02)	-0.02 (-0.07, 0.04)	-0.03 (-0.06, 0.00)	0.06 (-0.05, 0.16)

Abbreviations: ECG, electrocardiogram; Aβ, amyloid β; NfL, neurofilament light chain protein.
[a] The original data of the biomarkers were natural log-transformed due to skewed distributions.
[b] Plasma Aβ42/Aβ40 ratio was multiplied by 100.
[c] Models were adjusted for age, sex, education, resting heart rate (QT, JT and QRS intervals only), *APOE* genotype, smoking status, alcohol intake, body mass index, the number of chronic diseases, and use of anti-thrombotic agents, cardiac agents, and QT prolonging drugs.
**P*<0.05, †*P*<0.01.
